# The relationship between vitamin D and estimated glomerular filtration rate and urine microalbumin/creatinine ratio in Korean adults

**DOI:** 10.3164/jcbn.17-69

**Published:** 2017-11-28

**Authors:** Sung Gil Kim, Gwang Seok Kim, Jun Ho Lee, Ae Eun Moon, Hyun Yoon

**Affiliations:** 1 Department of Radiological Science, Hanlyo University, 94-13 Hallyeodae-gil, Gwangyang-eup, Gwangyang-si, Jeollanam-do 57764, Korea; 2 Department of Emergency Medical Technology, Chungbuk Health and Science University, 10 Deogam-gil, Naesu-eup, Cheongwon-gu, Cheongju-si, Chungcheongbuk-do 28150, Korea; 3 Department of Biomedical Laboratory Science, Wonkwang Health Science University, 345-13 Sinyong-dong, Iksan-si, Jeollabuk-do 54538, Korea; 4 Department of Dental Hygiene, Honam University, 417 Eodeung-daero, Gwangsan-gu, Gwangju 62399, Korea; 5 Department of Biomedical Laboratory Science, Hanlyo University, 94-13 Hallyeodae-gil, Gwangyang-eup, Gwangyang-si, Jeollanam-do 57764, Korea

**Keywords:** vitamin D, estimated glomerular filtration rate, urine microalbumin/creatinine ratio

## Abstract

The present study was conducted to assess the association between 25-hydroxyvitamin D [25(OH)D], estimated glomerular filtration rate (eGFR) and urine microalbumin/creatinine ratio (uACR) in Korean adults. Data on 4,948 adults aged ≥20 years from the Korean National Health and Nutrition Examination Survey V-3 (2012) were analyzed. After adjusting for the related variables (except age), the odds ratios (ORs) of vitamin D deficiency with the normal group as a reference were significantly higher in the decreased eGFR plus elevated uACR group [3.089 (95% CI, 1.722–5.544)], but not in the elevated uACR [1.247 (95% CI, 0.986–1.577)] and decreased eGFR group [1.303 (95% CI, 0.789–2.152)]. However, when further adjusting for age, the ORs of vitamin D deficiency with the normal group as a reference were significantly higher in the elevated uACR group [1.312 (95% CI, 1.035–1.662)], decreased eGFR group [1.761 (95% CI, 1.062–2.919)] and the decreased eGFR plus elevated uACR group [3.549 (95% CI, 1.975–6.365)]. In conclusion, vitamin D deficiency was positively associated with the elevated uACR and decreased eGFR. In addition, vitamin D level decreased greatly when decreased eGFR and elevated uACR appeared simultaneously.

## Introduction

Chronic kidney disease (CKD) is a global public health problem with 20 million adult Americans currently living with CKD in various stages of CKD: >400,000 individuals with end-stage kidney disease and >300,000 individuals requiring maintenance hemodialysis.^(1–3)^ CKD is defined by an estimated glomerular ﬁltration rate (eGFR) <60 ml/min/1.73 m^2^; a decrease in eGFR is a risk factor for cardiovascular disease (CVD) and is correlated with cardiovascular mortality and morbidity in high-risk groups.^(4,5)^ Albuminuria is a well-known predictor of CKD progression and is considered to be an early sign of glomerular damage used as a risk factor for end-stage renal disease in people with diabetes mellitus.^(6,7)^

Vitamin D from the diet or dermal synthesis from sunlight is biologically inactive [25-hydroxyvitamin D, 25(OH)D], which is metabolized to the biologically active 1,25 dihydroxyvitamin D [1,25(OH)_2_D] through enzymatic conversion in the kidney.^(8,9)^ 25(OH)D usually functions as storage due to its relatively long half-life of 2–3 weeks, and the total vitamin D status in the human body is generally estimated through measurements of serum 25(OH)D.^(10)^ Vitamin D is known to be involved in calcium and phosphate absorption in the intestines, and maintains sufficient concentrations of circulating calcium and phosphate levels and normal mineralization of bone by providing the minerals to bone-forming sites.^(11,12)^

Recently, vitamin D has receiving an attention on concerning its effect on CKD and CVD.^(13,14)^ It is important to monitor eGFR and the urine microalbumin/creatinine ratio (uACR) in patients with CKD and progressive CVD. In particular, when a decrease in eGFR is combined with an increase in uACR, CVD mortality rates in patients with CKD increase greatly.^(15)^ The Republic of Korea has recently become known as a country that has a severe vitamin D deficiency problem,^(16)^ and the burden of CKD and CVD are also increasing. Therefore, our objective in this study was to assess the association between vitamin D and eGFR and uACR in Korean adults using data from the fifth Korean National Health and Nutrition Examination Survey (KNHANES V-3; 2012) to be representative of the Korean population.

## Methods

### Study subjects

This study was performed using data from the Korean National Health and Nutrition Examination Survey (KNHANES V-3). KNHANES V-3 were each conducted for 1 years (2012), using a rolling sampling survey that involved a complex, stratified, multistage, probability cluster survey of a representative sample of the non-institutionalized civilian population in South Korea. The survey was composed of three parts: a health interview survey, a health examination survey and a nutrition survey. Each survey was conducted by specially trained interviewers. The interviewers were not provided with any prior information regarding specific participants before conducting the interviews. Participants provided written informed consent to participate in this survey, and we received the data in anonymized form. In the KNHANES V-3 (2012), 8,958 individuals over age 1 were sampled for the survey. Among them, of the 6,665 subjects who participated in the KNHANES V-3, we limited the analyses to adults aged ≥20 years. We excluded participants 1,717 subjects whose data were missing for important analytic variables, such as serum 25(OH)D, urine microalbumin and creatinine level, or various blood chemistry tests; pregnant women; and a high uACR (uACR; ≥3,000 mg/g) indicative of nephrotic-range albuminuria because a previous study found an association between altered vitamin D metabolism and nephrotic syndrome.^(17)^ Finally, 4,948 subjects were included in the statistical analysis. The KNHANES V-3 study has been conducted according to the principles expressed in the Declaration of Helsinki. (Institutional Review Board No, 2010-02CON-21-C). All participants in the survey signed an informed written consent form. Further information can be found in “The KNHANES V-3 (2012) Sample”, which is available on the KNHANES website. The official website of KNHANES (http://knhanes.cdc.go.kr) is currently operating an English-language information homepage. The data of the respective year are available to everyone at the free of charge. If the applicant enters simple subscription process and his/her email address in the official website of KNHANES, the data of the respective year can download to free of charge. If additional information is required, the readers can contact the department responsible for data (Su Yeon Park, sun4070@korea.kr).

### General characteristics and blood chemistry

 Research subjects were classified by sex (men or women), smoking (non-smoker or ex-smoker or current smoker), alcohol drinking (yes or no) and regular exercise (yes or no). In the smoking category, participants who smoked more than one cigarette a day, those who had previously smoked but do not presently smoke, and those who never smoked were classified into the current smoker, ex-smoker, and non-smoker groups, respectively. Alcohol drinking was indicated as “yes” for participants who had consumed at least one glass of alcohol every month over the last year. Regular exercise was indicated as “yes” for participants who had exercised on a regular basis regardless of indoor or outdoor exercise. (Regular exercises was defined as 30 min at a time and 5 times/weeks in the case of moderate exercise, such as swimming slowly, doubles tennis, volleyball, badminton, table tennis and carrying light objects; and for 20 min at a time and 3 times/weeks in the case of vigorous exercise, such as running, climbing, cycling fast, swimming fast, football, basketball, jump rope, squash, singles tennis and carrying heavy objects). Anthropometric measurements included measurement of body mass index (BMI) and waist measurement (WM), as well as final measurements of systolic blood pressure (SBP) and diastolic blood pressure (DBP). Blood chemistries included measurements of total cholesterol (TC), high density lipoprotein cholesterol (HDL-C), triglycerides (TGs), fasting blood glucose (FBG), blood urea nitrogen (BUN), serum creatinine (Crea), urine microalbumin, urine creatinine and 25-hydroxyvitamin D.

### Glomerular filtration rate and urine microalbumin/creatinine ratio and serum 25(OH)D

Glomerular filtration rate (GFR) was estimated from the simplified equation developed using MDRD data: eGFR = 186.3 × (serum creatinine in mg/dl)^−1.154^ × age^−0.203^ × (0.742 for women) × (1.212 if African American).^(18)^ Decreased eGFR was classified as eGFR <60 ml/min/1.73 m^2^. Urine microalbumin was measured with a turbidimetric assay (Albumin; Roche, Germany) using a Hitachi Automatic Analyzer 7600 (Hitachi, Japan). The urine creatinine was measured with a colorimetric assay (CREA; Roche, Indianapolis, IN) using a Hitachi Automatic Analyzer 7600. Elevated uACR was classified as uACR ≥30 mg/g. Serum 25(OH)D levels were measured with a radioimmunoassay (25-hydroxy-vitamin D ^125^I RIA Kit; DiaSorin, Still Water, MN) using a 1470 Wizard Gamma Counter (Perkin Elmer, Turku, Finland). To minimize the analytical variation, serum 25(OH)D levels were analyzed by the same institute, which carried out a quality assurance program through the analysis period. Serum 25(OH)D levels were classified as either vitamin D deficiency [25(OH)D <15.0 ng/ml] and vitamin D sufficiency [25(OH)D ≥15.0 ng/ml].^(19)^

### Statistical analysis

The collected data were statistically analyzed using SPSS WIN ver. 18.0 (SPSS Inc., Chicago, IL). The distributions of the participant characteristics were converted into percentages, and the successive data were presented as averages with standard deviations. The distribution and average difference in clinical characteristics according to vitamin D sufficiency and vitamin D deficiency were calculated using chi-squared and an independent *t* test. In the case of the analysis of covariance test (ANCOVA) for the serum 25(OH)D, the 2 models constructed were: 1) adjusted for alcohol drinking, SBP, DBP, BMI and WM; 3) further adjusted for TC, TGs, HDL-C, BUN and FBG; 2) further adjusted for age. In the case of logistic regression for odds ratio of vitamin D deficiency, the 4 models constructed were: 1) non-adjusted; 2) adjusted for alcohol drinking, SBP, DBP, BMI and WM; 3) further adjusted for TC, TGs, HDL-C, BUN and FBG; 4) further adjusted for age. The significance level for all of the statistical data was set as *p*<0.05.

## Results

### Clinical characteristics of the research subjects

 The clinical characteristics of the research subjects are shown in Table 1. Serum 25(OH)D, eGFR and uACR were 20.38 ± 4.58 ng/dl, 89.78 ± 17.01 ml/min/1.73 m^2^ and 19.30 ± 102.13 mg/g, respectively, in subjects with vitamin D sufficiency (*n* = 3,034). The prevalence rates of decreased eGFR and elevated uACR were 3.5% (*n* = 107) and 8.4% (*n* = 254), respectively. Serum 25(OH)D, eGFR and uACR were 11.92 ± 2.15 ng/dl, 94.75 ± 19.14 ml/min/1.73 m^2^ and 21.39 ± 130.56 mg/g, respectively, in subjects with vitamin D deficiency (*n* = 1,914). The prevalence rates of decreased eGFR and elevated uACR were 2.7% (*n* = 51) and 8.9% (*n* = 170), respectively.

### Clinical characteristics of the subjects according to decreased eGFR, elevated uACR and decreased GFR plus elevated uACR

Clinical characteristics of the subjects according to decreased eGFR and elevated uACR are shown in Table 2. eGFR and uACR were 93.26 ± 16.44 ml/min/1.73 m^2^ and 5.48 ± 5.46 mg/g for the normal group, 92.69 ± 17.60 ml/min/1.73 m^2^ and 132.28 ± 204.03 mg/g for the elevated uACR group, 52.78 ± 6.75 ml/min/1.73 m^2^ and 10.38 ± 7.51 mg/g for the decreased eGFR group, and 46.85 ± 12.91 ml/min/1.73 m^2^ and 411.93 ± 726.23 mg/g for the decreased eGFR plus elevated uACR group, respectively. Variables with significant difference in normal, elevated uACR, decreased eGFR and decreased GFR plus elevated uACR group are current drink (*p*<0.001), SBP (*p*<0.001), DBP (*p*<0.001), BMI (*p*<0.001), WM (*p*<0.001), TGs (*p*<0.001), HDL-C (*p*<0.001), BUN (*p*<0.001), Crea (*p*<0.001), FBG (*p*<0.001) and age (*p*<0.001). However, gender (*p* = 0.373), current smoke (*p* = 0.249), regular exercise (*p* = 0.112) and TC (*p* = 0.807) were not significant.

### Comparison of 25(OH)D levels and odds ratios of vitamin D deficiency according to decreased eGFR, elevated uACR and decreased GFR plus elevated uACR

Comparison of odds ratios (ORs) of vitamin D deficiency according to decreased eGFR, elevated uACR and decreased GFR plus elevated uACR are shown in Table 3 and 4. After adjusting for the related variables (except age), the ORs of vitamin D deficiency with the normal group as a reference were significantly higher in the decreased eGFR plus elevated uACR group [3.089 (95% CI, 1.722–5.544)], but not in the elevated uACR [1.247 (95% CI, 0.986–1.577)] and decreased eGFR group [1.303 (95% CI, 0.789–2.152)]. However, when further adjusting for age, the ORs of vitamin D deficiency with the normal group as a reference were significantly higher in the elevated uACR group [1.312 (95% CI, 1.035–1.662)], decreased eGFR group [1.761 (95% CI, 1.062–2.919)] and decreased eGFR plus elevated uACR group [3.549 (95% CI, 1.975–6.365)]. 25(OH)D levels (M ± SE) were 17.20 ± 0.08 ng/dl (95% CI, 17.04–17.36) for the normal group, 16.62 ± 0.30 ng/dl (95% CI, 16.04–17.21) for the elevated uACR group, 16.41 ± 0.60 ng/dl (95% CI, 15.23–17.59) for the decreased eGFR group and 13.82 ± 0.75 ng/dl (95% CI, 12.34–15.29) for the decreased eGFR plus elevated uACR group (*p*<0.001) (Table 4).

## Discussion

In the present study, an investigation into the association between vitamin D and eGFR and uACR in Korean adults was carried out using data from the KNHANES V-3 conducted in 2012. Vitamin D deficiency was positively associated with the elevated uACR, and decreased eGFR and vitamin D level decreased greatly when decreased eGFR and elevated uACR appeared simultaneously.

**Fig. 1:**
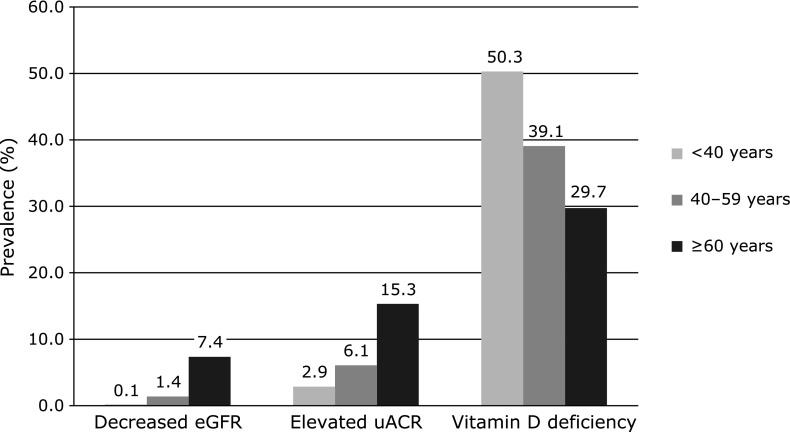
Comparisons of the vitamin D deficiency, decreased eGFR, and elevated uACR according to age. Decreased eGFR: eGFR <60 ml/min/1.73 m^2^; Elevated uACR: uACR ≥30 mg/g; vitamin D deficiency: 25(OH)D <15.0 ng/dl. The prevalence of elevated uACR (*p*<0.001) and decreased eGFR (*p*<0.001) levels were increased as an increase of age, but the prevalence of vitamin D deficiency (*p*<0.001) was decreased as an increase of age.

Vitamin D deficiency is found in various populations worldwide in high proportions and was associated with diabetes, hypertension and insulin resistance.^(20,21)^ In particular, vitamin D deficiency patients with CKD have been associated with a higher risk of cardiovascular events and mortality and accelerate a progression of kidney disease.^(22,23)^ Ravani *et al.*^(23)^ suggested that serum 25(OH)D is an independent inverse predictor of renal disease progression and death in patients with earlier stages of CKD. However, among the research on the association between vitamin D and eGFR or uACR, previous results have been inconsistent. Park *et al.*^(24)^ reported that 25(OH)D was positively associated with eGFR (*p*<0.001) and negatively associated with uACR (*p* = 0.043) in Korean adults. In contrast, O'Seaghdha *et al.*^(25)^ reported that 25(OH)D was not associated with either eGFR (*p*_trend_ = 0.3) or uACR (*p*_trend_ = 0.9) in the Framingham Heart Study. In the present study, after adjusting for the related variables (except age), the association between vitamin D and elevated uACR and decreased eGFR group was not significant, and these results were similar to the study of O'Seaghdha *et al.* However, when further adjusting for age, the ORs of vitamin D deficiency with the normal group as a reference were significantly higher in the elevated uACR group [1.312 (95% CI, 1.035–1.662)] and decreased eGFR group [1.761 (95% CI, 1.062–2.919)], and these results were similar to the study of Park *et al.* Age is a strong risk factor of albuminuria and CKD.^(26,27)^ In our results, the prevalence of elevated uACR and decreased eGFR levels were increased as an increase of age, but the prevalence of vitamin D deficiency was decreased (Fig. 1). Vitamin D was increased as an increase of the age because the outdoor activity in the Korean elderly is higher than in the younger.^(28)^ However, aging affects the formation of 1,25(OH)_2_D, the active form of vitamin D. Although vitamin D [25(OH)D] increases, production of 1,25(OH)_2_D is reduced by 50% as a result of a decline in renal function according to increase of age.^(29)^ Therefore, some studies emphasized that need to measure both 25(OH)D and 1,25(OH)_2_D in vitamin D deficiency.^(10,30,31)^

We examined the ORs of vitamin D deficiency when the decreased eGFR and elevated uACR occurred simultaneously. It is important to monitor uACR levels in populations with CKD. Albuminuria is an unequivocal surrogate marker for CKD progression as well as future cardiovascular events and its reduction is used as a treatment goal for these diseases.^(32)^ In addition, albuminuria is an early warning sign of diabetic nephropathy (DN), and DN is associated with an elevated risk of progression toward ESRD as well as increase of cardiovascular events and mortality.^(33–35)^ In the present study, the ORs of vitamin D deficiency in the decreased eGFR plus elevated uACR group [3.549 (95% CI, 1.975–6.365)] was very higher than the elevated uACR group [1.312 (95% CI, 1.035–1.662)] or decreased eGFR group [1.761 (95% CI, 1.062–2.919)]. We thought these results that the synergistic interaction between the decreased eGFR and elevated uACR. Levey *et al.*^(18)^ suggested that the synergistic interaction between the decreased eGFR and elevated uACR. They reported that the progressive CKD in the decreased eGFR plus elevated uACR group (at least 9.4 times, up to 57 times) was higher than the elevated uACR group (at least 0.4 times, up to 8.1 times). In particular, the OR of end stage renal disease (ESRD) for the decreased eGFR plus elevated uACR group (at least 40 times, up to 2,286 times) was greatly higher than the elevated uACR group (at least 3.8 times, up to 67 times).

Vitamin D from the diet or skin synthesis is biologically inactive and is converted to 25(OH)D in the liver. And then, 25(OH)D is further hydroxylated in the kidneys to form 1,25(OH)_2_D which is the biologically active form of vitamin D.^(36)^ However, in patients with CKD, there is high rate of prevalence of vitamin D deficiency because the reduced ability to convert the active form 1,25(OH)_2_D.^(37)^ Therefore, if renal function decreases rapidly by the synergistic interaction between the decreased eGFR and elevated uACR, the frequency of vitamin D deficiency may increase. On the other hand, renal function may decrease as vitamin D deficiency. Vitamin D is known to suppress the renin gene transcription,^(38)^ and administration of vitamin D preparations such as calcitriol and paricalcitol inhibit renin expression and consequently reduce angiotensin II expression.^(39)^ Angiotensin II is a key mediator of proteinuria, raises efferent glomerular arteriole resistance and induces transforming growth factor (TGF)-β1, which inhibits cell proliferation and increases apoptosis in the kidney,^(40,41)^ and so the reduction of angiotensin II by vitamin D may be a mechanism to counter these effects. NF-κB is involved in the regulation of inflammatory cytokines that may promote inflammation and fibrogenesis in kidney disease.^(42)^ In mice with obstructive nephropathy, administration of paricalcitol was found to block NF-κB and attenuate tubule-interstitial inflammation.^(43)^ Cohen-Lahav *et al.*^(44)^ reported that vitamin D upregulates IkappaBalpha (IκBα) levels by increasing mRNA stability; an increase in IκBα levels reduces nuclear translocation of NF-κB and thereby downgrades its activity. It is unclear whether the decrease of renal function increased the incidence of vitamin D deficiency, or vitamin D deficiency decreases the renal function. Furthermore, the association between the decrease of renal function and vitamin D is still debated. In the relationship between vitamin D and CKD and albuminuria, the result may differ according to the country and ethnicity, study population and the use of different reference GFR methods. In Asian, MDRD and CKD-EPI Equations for Taiwanese and Japanese adults is modified for their studied population. However, there is no definite modified model for Korean adults yet. Therefore, research is necessary to modify the MDRD and CKD-EPI Equations for the Korean adults.

There are a few limitations in the present study. First, season is the most important determinant of serum 25(OH)D levels, but the data of the KNHANES V-3 study did not specify serum 25(OH)D levels according to season. Second, serum calcium concentration and daily intake of vitamin D are important determinants of serum 25(OH)D levels, but these were not measured as part of the KNHANES V-3 study. Therefore, serum calcium concentration and daily intake volume of vitamin D could not be used as an adjustment variable. Third, parathyroid hormone (PTH) is an important determinant of serum vitamin D levels as increased PTH promotes calcium influx into adipocytes, where intracellular calcium enhances lipogenesis.^(45)^ Therefore, serum vitamin D levels could change depending on serum PTH. However, in the data from the KNHANES V-3 study, there are no measurements of PTH of the participants (adults ≥20 years of age). The serum 25 (OH)D levels for each season, along with calcium and PTH levels, should be included as variables of vitamin D status in future studies. Fourth, because this was a cross-sectional study, the ability to establish a causal relationship between vitamin D and uACR and eGFR was limited. Therefore, more accurate results might be obtained by performing a cohort study by adding these variables.

## Conclusion

The present study investigated the association between serum 25(OH)D and urine microalbumin/creatinine ratio and estimated glomerular filtration rate in Korean adults using data from the KNHANES V-3 conducted in 2012. Vitamin D deficiency was positively associated with the elevated uACR and decreased eGFR. In addition, vitamin D level decreased greatly when decreased eGFR and elevated uACR appeared simultaneously.

## Figures and Tables

**Table 1:** Clinical characteristics of the research subjects

*n* (%), Mean ± SD					
Variables	Category	Total (*n* = 4,948)	Vit. D sufficiency (*n* = 3,034)	Vit. D deficiency (*n* = 1,914)	*p* value
Age (year)		51.88 ± 16.02	54.19 ± 15.38	48.21 ± 16.34	<0.001
	<40	1,293 (26.1)	642 (21.1)	651 (34.0)	
	40–59	1,883 (38.1)	1,146 (37.8)	737 (38.5)	
	≥60	1,772 (35.8)	1,246 (41.1)	526 (27.5)	
Gender	Men	2,714 (43.9)	1,490 (49.1)	674 (35.7)	<0.001
Drinking	Current drinker	2,495 (50.4)	1,574 (51.9)	921 (48.1)	0.005
Smoking	Current smoker	1,020 (20.6)	729 (24.0)	291 (15.2)	<0.001
Exercising	Regular exerciser	313 (6.3)	218 (7.2)	95 (5.0)	0.001
^α^eGFR (ml/min/1.73 m^2^)		91.70 ± 18.02	89.78 ± 17.01	94.75 ± 19.14	<0.001
	eGFR ≥60	4,790 (96.8)	2,927 (96.5)	1,863 (97.3)	0.097
	eGFR <60	158 (3.2)	107 (3.5)	51 (2.7)	
^β^uACR (mg/g)		20.11 ± 113.96	19.30 ± 102.13	21.39 ± 130.56	<0.001
	uACR <30	4,524 (91.4)	2,780 (91.6)	1,744 (91.1)	0.529
	uACR ≥30	424 (8.6)	254 (8.4)	170 (8.9)	
^γ^BMI (kg/m^2^)		23.84 ± 3.34	23.89 ± 3.18	23.76 ± 3.58	0.181
^δ^WM (cm)		81.50 ± 9.58	82.09 ± 9.12	80.57 ± 10.21	<0.001
^ε^SBP (mmHg)		120.08 ± 16.98	120.72 ± 16.70	119.07 ± 17.36	0.001
^ζ^DBP (mmHg)		75.91 ± 10.49	75.89 ± 10.33	75.94 ± 10.73	0.86
^η^TC (mg/dl)		190.51 ± 36.11	191.94 ± 36.26	188.23 ± 35.76	<0.001
^θ^TGs (mg/dl)		131.13 ± 87.19	130.88 ± 82.74	131.51 ± 93.13	0.805
^κ^HDL-C (mg/dl)		51.52 ± 12.63	51.30 ± 12.32	51.88 ± 13.10	0.113
^λ^FBG (mg/dl)		98.92 ± 21.78	99.53 ± 21.61	97.95 ± 22.02	0.013
^µ^BUN (mg/dl)		14.65 ± 4.49	15.24 ± 4.42	13.72 ± 4.44	<0.001
^π^Crea (mg/dl)		0.84 ± 0.23	0.86 ± 0.21	0.82 ± 0.27	<0.001
^σ^25(OH)D (ng/dl)		17.11 ± 5.62	20.38 ± 4.58	11.92 ± 2.15	<0.001
Urine microalbumin (µg/dl)		23.71 ± 113.79	22.08 ± 98.68	26.31 ± 134.29	0.203
Urine creatinine (mg/dl)		150.10 ± 84.99	144.78 ± 79.89	158.52 ± 91.89	<0.001

^α^eGFR, estimated glomerular filtration rate; ^β^uACR, urine microalbumin/creatinine ratio; ^γ^BMI, body mass index; ^δ^WM, waist measurement; ^ε^SBP, systolic blood pressure; ^ζ^DBP, diastolic blood pressure; ^η^TC, total cholesterol; ^θ^TGs, triglycerides; ^κ^HDL-C, high density lipoprotein cholesterol; ^λ^FBG, fasting blood glucose; ^µ^BUN, blood urea nitrogen; ^π^Crea, serum creatinine; ^σ^25(OH)D, 25-hydroxyvitamin D. Vit. D sufficiency: 25(OH)D ≥15.0 ng/dl; Vit. D deficiency: 25(OH)D <15.0 ng/dl.

**Table 2:** Clinical characteristics of the subjects according to decreased eGFR, elevated uACR and decreased eGFR plus elevated uACR

(*n* = 4,948)					
Variables	^α^Normal (*n* = 4,431)	^β^Elevated uACR (*n* =359)	^γ^Decreased eGFR (*n* = 93)	^ζ^Decreased eGFR plus Elevated uACR (*n* = 65)	*p* value
25(OH)D (ng/dl)	17.07 ± 5.56	17.07 ± 5.96	19.05 ± 6.55	17.06 ± 6.39	0.01
Vit. D deficiency	1,718 (38.8)	145 (40.4)	26 (28.0)	25 (38.5)	0.174
Men	1,957 (44.2)	143 (39.8)	43 (46.2)	31 (47.7)	0.373
Current drinker	2,287 (51.6)	160 (44.6)	33 (35.5)	15 (23.1)	<0.001
Current smoker	901 (20.3)	75 (20.9)	24 (25.8)	20 (30.8)	0.249
Regular exerciser	268 (6.0)	33 (9.2)	7 (7.5)	5 (7.7)	0.112
Age (year)	50.51 ± 15.68	60.75 ± 14.55	71.51 ± 8.48	67.97 ± 12.42	<0.001
SBP (mmHg)	118.74 ± 16.21	132.44 ± 18.55	128.95 ± 18.03	130.46 ± 22.47	<0.001
DBP (mmHg)	75.72 ± 10.19	79.03 ± 12.16	73.69 ± 10.13	74.34 ± 16.57	<0.001
BMI (kg/m^2^)	23.71 ± 3.28	25.15 ± 3.69	24.05 ± 3.24	24.86 ± 3.75	<0.001
WM (cm)	81.06 ± 9.47	85.56 ± 9.92	83.61 ± 8.89	86.00 ± 9.46	<0.001
TC (mg/dl)	190.65 ± 35.71	189.34 ± 39.93	190.40 ± 37.74	187.25 ± 39.22	0.807
TGs (mg/dl)	128.43 ± 85.32	157.14 ± 101.83	135.76 ± 79.53	164.45 ± 106.26	<0.001
HDL-C (mg/dl)	51.90 ± 12.70	48.77 ± 11.22	47.77 ± 11.67	46.25 ± 12.45	<0.001
FBG (mg/dl)	97.35 ± 16.45	114.16 ± 35.01	103.40 ± 17.81	115.69 ± 37.48	<0.001
eGFR (ml/min/1.73 m^2^)	93.26 ± 16.44	90.69 ± 17.60	52.78 ± 6.75	46.85 ± 12.91	<0.001
uACR (mg/g)	5.48 ± 5.46	132.28 ± 204.03	10.38 ± 7.51	411.93 ± 726.23	<0.001
BUN (mg/dl)	14.28 ± 3.92	15.60 ± 4.36	21.03 ± 6.77	25.63 ± 11.29	<0.001
Crea (mg/dl)	0.83 ± 0.16	0.81 ± 0.17	1.27 ± 0.30	1.63 ± 1.08	<0.001

^α^Normal: eGFR ≥60 ml/min/1.73 m^2^ and uACR ≤30 mg/g. ^β^Elevated uACR: uACR ≥30 mg/g, ^γ^Decreased eGFR: eGFR <60 ml/min/1.73 m^2^, ^ζ^Decreased eGFR plus Elevated uACR: eGFR <60 ml/min/1.73 m^2^ and uACR ≥30 mg/g.

**Table 3:** Comparisons of vitamin D deficiency odds ratios according to decreased eGFR, elevated uACR and decreased eGFR plus elevated uACR

(*n* = 4,948)					
Variables	Category	Vit. D deficiency [25(OH)D <15.0 ng/dl]			
		Model 1	Model 2	Model 3	Model 4
Normal	eGFR ≥60 and uACR <30	1	1	1	1
Elevated uACR	uACR ≥30	1.070 (0.859–1.332)	1.208 (0.961–1.517)	1.247 (0.986–1.577)	1.312 (1.035–1.662)
Decreased eGFR	eGFR <60	0.613 (0.388–0.968)	0.709 (0.445–1.127)	1.303 (0.789–2.152)	1.761 (1.062–2.919)
Decreased eGFR plus Elevated uACR	eGFR <60 and uACR ≥30	0.987 (0.597–1.633)	1.172 (0.702–1.959)	3.089 (1.722–5.544)	3.549 (1.975–6.365)

25(OH)D, 25-hydroxyvitamin D; Vit. D deficiency, 25(OH)D <15.0 ng/dl; eGFR, estimated glomerular filtration rate; uACR, urine microalbumin/creatinine ratio. Model 1 [odds ratio (OR), 95% CI)], Non-adjusted; Model 2 [OR, 95% CI], adjusted for alcohol drinking, SBP, DBP, BMI and WM; Model 3 [OR, 95% CI], Model 2 further adjusted for TGs, HDL-C, BUN and FBG; Model 4 [OR, 95% CI], Model 3 further adjusted for age.

**Table 4:** Comparisons of 25(OH)D levels according to decreased eGFR, elevated uACR and decreased eGFR plus elevated uACR

(*n* = 4,948)				
Variables	Category	25(OH)D levels (ng/dl)		
		Model 1	Model 2	Model 3
Normal	eGFR ≥60 and uACR <30	17.11 ± 0.08 (16.95–17.28)	17.17 ± 0.08 (17.01–17.33)	17.20 ± 0.08 (17.04–17.36)
Elevated uACR	uACR ≥30	16.78 ± 0.30 (16.19–17.37)	16.75 ± 0.30 (16.15–17.34)	16.62 ± 0.30 (16.04–17.21)
Decreased eGFR	eGFR <60	18.58 ± 0.58 (17.44–19.72)	17.20 ± 0.61 (16.01–18.40)	16.41 ± 0.60 (15.23–17.59)
Decreased eGFR plus Elevated uACR	eGFR <60 and uACR ≥30	16.55 ± 0.70 (15.18–17.91)	14.18 ± 0.76 (12.68–18.40)	13.82 ± 0.75 (12.34–15.29)
	*p* value	0.039	0.001	<0.001

25(OH)D, 25-hydroxyvitamin D; eGFR, estimated glomerular filtration rate; uACR, urine microalbumin/creatinine ratio. Model 1 (Mean ± SE, 95% CI), alcohol drinking, SBP, DBP, BMI and WM; Model 2 (Mean ± SE, 95% CI), Model 1 further adjusted for TGs, HDL-C, BUN and FBG; Model 3 (Mean ± SE, 95% CI), Model 2 further adjusted for age.
